# Microencapsulation Curcuminoids for Effective Delivery in Pharmaceutical Application

**DOI:** 10.3390/pharmaceutics11090451

**Published:** 2019-09-02

**Authors:** Lee Fung Ang, Yusrida Darwis, Lip Yee Por, Mun Fei Yam

**Affiliations:** 1 School of Pharmaceutical Sciences, Universiti Sains Malaysia, Pulau Pinang 11800, Malaysia; 2 Department of Computer System and Technology, Faculty of Computer Science and Information Technology, University of Malaya, Kuala Lumpur 50603, Malaysia

**Keywords:** gelatin B, chitosan, microencapsulation, curcuminoid

## Abstract

Curcuminoids have been long proven to possess antioxidant, anti-inflammatory and antibacterial properties which are crucial in their role as a pharmacological active agent. However, its poor solubility, high oxidative degradation, light sensitivity and poor bioavailability have been huge hurdles that need to be overcome for it to be administered as an oral or even a topical medication. In this present study, a complex coacervation microencapsulation approach was used to encapsulate the curcuminoids using both gelatin B and chitosan (at the optimum ratio of 30:1% *w*/*w*) for a more efficient drug delivery system. Curcuminoids microcapsules (CPM) were developed to be spherical in shape, discrete and free flowing with a reduced color staining effect. The thick wall of the CPM contributes directly to its integrity and stability. Cross-linking increases the density of polymers’ wall network, hence, further increasing the decomposition temperature of curcuminoids microcapsules. Microencapsulation demonstrated an increment in curcuminoids solubility, while chemical cross-linking allowed for sustained release of the drug from the microcapsules by lowering the swelling rate of the available polymer networks. Thus, the microcapsules complied with the zero order release kinetics with super case-II transport mechanism. On the basis of all that was discussed above, it can be safely concluded that CPM should be incorporated in delivery system of curcuminoid, especially in its topical delivery for controlled drug release purposes, for not only a more efficient drug delivery system design but also a more efficacious optimization of the pharmacological benefits of curcuminoids.

## 1. Introduction

Curcuminoids, the extract obtained from the rhizome of turmeric is a herb belonging to the ginger family, and its contents consists mainly of curcumin (~77%), demethoxycurcumin (~17%) and bisdemethoxycurcumin (~3%) [1]. The biological characteristics of curcuminoids have been scientifically investigated since the mid-twentieth century [2]. Curcumin and its derivatives are typical flavonoid compounds that exhibit a wide range of pharmacological activities, such as anti-cancer [3,4,5], antioxidant [6], anti-angiogenic [7,8], antibacterial [9], anti-inflammatory [10], antiviral and antifungal [11] properties. However, due to its poor solubility and bioavailability, oxidative degradation, light sensitivity, rapid metabolism, systemic clearance and rapid hydrolysis in alkaline solutions, there are huge limitations for their usage in pre-clinical and clinical models.

Gelatin is biodegradable, non-toxic, and easy to crosslink and to modify chemically. It is frequently used in food, pharmaceutical, biomedical and photographic industries. It is commonly used as the main ingredient of hard and soft capsules, microspheres, sealants for vascular prostheses, and wound dressings and adsorbent pads for surgical use and tissue regeneration. Gelatin is soluble in aqueous solutions at a temperature of about 40 °C and present in the sol state [12]. When gelatin is cooled below 30–35 °C, the random coil polypeptide chains will undergo a conformational disorder-order transition and partly regenerate the collagen triple-helix structure and form thermos reversible gels by associating helices in junction zones stabilized by hydrogen bonds [12,13]. Since gelatin is soluble in aqueous solutions, gelatin used as coating material or a matrix of microcapsule products must be submitted to cross-linking, which improves both the thermal and mechanical stability of the biopolymer [12]. Cross-linking is important for the gelatin capsules to be insoluble at high temperatures, to reduce swelling in water and decrease permeability across cell membranes. Formaldehyde and glutaraldehyde are the most common agents used in gelatin cross-linking. 

Gelatin is widely used as the main ingredient of the complex coacervation in the food and pharmaceutical industries [14,15,16,17,18,19]. The most important property of this protein is that gelatin is a polyampholyte with amine (–NH_2_) and carboxyl (–COOH) functional groups along with hydrophobic groups. Gelatin is a random coil polymer carrying positively- and negatively-charged sites in almost a 1:1 ratio. Additionally, it is associated with a small persistent length of about 2 nm [20]. In solution, gelatin can be ionized to either –COO^−^ or –NH_3_^+^ depending on the pH of the solution. At pH above the isoelectric point, gelatin charged negatively; whereas at pH below the isoelectric point, gelatin charged positively. Hence, gelatin will react with oppositely charged biomolecules in the same medium via electrostatic interaction to form polyionic complexes [21]. For example, in a mixture containing gelatin (a protein) and an anionic polysaccharide, adjustment of the pH to below the isoelectric point (pI) or the electrical equivalence pH (IEP) of gelatin would result in maximum electrostatic attraction where the two biopolymers are oppositely charged. For instance, complex coacervation occurs in a mixture of gum arabic (acacia) and type A gelatin at pH 4.5 [22], or in a mixture of gum arabic (acacia) and type B gelatin at pH 3.5. On the other hand, a gelatin-cationic polysaccharide system requires adjustment of the mixture pH to above the isoelectric point of gelatin for electrostatic attraction. For instance, complex coacervation occurred in a type B gelatin-chitosan system at pH 5.25–5.5 [23]. At pH 5.25–5.5, gelatin is negatively charged (since the isoelectric point of gelatin B is 4.7–5.2) and it will electrostatically interact with positively-charged chitosan to form an insoluble complex (complex coacervate). At such pH, gelatin-chitosan complex coacervation should be limited to type B gelatin. The isoelectric point of type A gelatin is typically pH 8–9, so its complex coacervation with chitosan should be limited to pH values above this. However, chitosan is precipitated at a solution of pH higher than 6 since the pKa of chitosan is about pH 6.3 [24].

Chitosan is widely used in the food, cosmetic and pharmaceutical industries as a carrier for drug delivery because it possesses excellent properties, such as non-toxicity, biodegradability, biocompatibility and absorption properties [24]. Additionally, a large number of amine groups provide availability of chitosan interacting with other substances [25]. Since the 1990s, chitosan microspheres have been used as controlled drug delivery systems for conventional drugs, protein drugs and bioactive compounds. Chitosan is particularly useful as coating material in microencapsulation for the controlled release of bioactive compounds. Chitosan can establish covalent or ionic bonds with cross-linking agents, for example, glutaraldehyde which forms Schiff bases with amino groups building a three-dimensional network; this increases the internal surface area for absorption and the encapsulated active substance stays retained, consequently as a controlled release carrier [25,26]. On the other hand, chitosan and its derivatives in microencapsulation of peptides and proteins have proven to enhance their permeation, since they have affinity for enzymes that usually degrade the peptide (e.g., insulin). Hence, the bioavailability of peptide drugs is increased due to permeation enhancement by chitosan, its enzyme inhibition and mucoadhesive properties [24]. Chitosan is a good coating material candidate for different microencapsulation techniques because its cationic properties in acidic conditions make it able to electrostatically interact with negatively charged molecules or polymers [27]. Chitosan can form nanoparticles by ionic gelation with polyphosphates and with nucleic acids [27]. In addition, chitosan is used for simple coacervation together with incompatible material, such as sodium sulfate. Simple coacervation is a simple and mild method, however, it results in low encapsulation efficiency [24]. Chitosan is also used for complex coacervation with type B gelatin since one behaves cationically and the other behaves anionically in solutions with pH value above the isoelectric point of gelatin. In addition, complex coacervation between chitosan and the negatively-charged lipid, lecithin, produces nanoparticles. Sodium alginate-chitosan systems have been widely studied. The release of albumin microencapsulated in this system is influenced by the molecular weight of chitosan used [24].

Microencapsulation is widely used in industry, pharmaceutical, agricultural and food as a technological advantage to assist with immobilization or entrapment, protection, controlled release, structuration and functionalization of active ingredient(s) [28]. Multiple studies have shown that curcumin microcapsules exhibit better stability against alkaline pH, light, oxygen and heat compared to its non-encapsulated form [29,30]. Microencapsulation technologies also possess the controlled release properties of active ingredients, thus improving the bioavailability of delivered active ingredients [31,32,33], allowing a more steady level of therapeutic drugs in the body for a longer period of time.

Microencapsulation can be achieved via different techniques, such as spray drying, spray cooling and spray chilling, spinning disk and centrifugal co-extrusion, extrusion, fluidized bed, coacervation, alginate beads, liposomes, RESS (rapid expansion of supercritical solutions) and inclusion encapsulation [34,35].

Coacervation encapsulation is a physiochemical process that can be classified as simple or complex coacervation. A simple coacervation system only contains one colloidal solute (e.g., only gelatin), while in the complex coacervation system there is more than one solute (e.g., gelatin and gum acacia) [36]. Complex coacervation can be explained as the separation of a macromolecular solution that iscomposed of two oppositely charged macro-ions into two immiscible liquid phases; the dense coacervate phase, which is relatively concentrated in macromolecules and the dilute equilibrium phase [37]. This is a unique and promising technology because of its very high achievable payload (up to 99%) and several characteristics, such as deliverability, stability, miscibility and controlled release property of its core ingredients [35]. The objectives of this study were to prepare and characterize microcapsules of curcuminoids using type B gelatin and chitosan by complex coacervation technique. Characteristics such as encapsulation efficiency, morphology and size, stability of microcapsules and the in vitro release profile of curcuminoids microcapsules have also been studied.

## 2. Materials and Methodology

### 2.1. Materials

Gelatin (Type B, Bloom No. 225), Tween 80 and phosphate buffered saline tablets were purchased from Sigma, St. Louis, MO, United States. Chitosan (molecular weight of 100,000–300,000), curcuminoids (mixture of curcumin, demethoxycurcumin and bisdemethoxycurcumin, >98%) and ethanol were obtained from Acros Organics, Morris Plains, NJ, United States. Formaldehyde 37–41%, 1-butanol and spectra grade potassium bromide were bought from Fisher Scientific, Bishop Meadow Rd, Loughborough, United Kingdom, while the HPLC grade methanol and acetonitrile were procured from J. T. Baker, Phillipsburg, NJ, United States. Citric acid anhydrous, di-sodium sulfate and di-sodium hydrogen phosphate (dihydrate) were bought from R & M Chemicals, Wardour Street, London, United Kingdom. Sodium hydroxide and glacial acetic acid were purchased from QRëC, Rawang, Selangor, Malaysia, respectively. Cellulose nitrate membrane filters (ø 13 mm, pore size 0.45 µm) were obtained from Whatman GmbH, Hahnestraße, Dassel, Germany.

### 2.2. Methods

#### 2.2.1. Complex Coacervation Microencapsulation

Gelatin B and chitosan were both dissolved in 1% *w*/*w* acetic acid in their respective containers. Curcuminoids (0.2 g) were first suspended in Tween 80, and then dispersed into 20 mL of chitosan solution via mechanical stirring of 1000 rpm at 50 °C for 30 min. After that, 20 mL of gelatin solution was added to the dispersed solution of curcuminoids at the rate of 1 mL/min using a syringe pump (Green Stream^®^ SY-P Argus 600, Heimberg, Switzerland) under constant stirring of 500 rpm and 50 °C (IKA^®^ Werke GmbH, Staufen, Breisgau, Germany). The mixture was then mixed homogeneously. The total polymer concentration was 2.55% *w*/*w* with gelatin to chitosan mixing ratio of 30:1% *w*/*w*. Thereafter, pH of the colloid was slowly adjusted to 5.5 by addition of 1 M sodium hydroxide solution (NaOH) gradually. Stirring of the colloid continued at the speed of 500 rpm for another 4 h at a standard 50 °C to induce coacervation. Thereafter, the liquid coacervate was cooled gradually to room temperature, and the temperature was then abruptly lowered to <10 °C by incubating the system in an ice-bath under constant stirring for 1 h. Subsequently, formaldehyde (2.5% *v*/*v* of formaldehyde the total colloid volume) was added drop by drop into the system and stirred for 30 min to produce a covalent cross-linked microcapsule. On the other hand, non-cross-linked microcapsules were prepared for the purpose of comparison. The drug loaded coacervate was washed with ethanol 3 times; the final time with cold distilled water and centrifugation was done at 1000 rpm for 5 min at 10 °C. Then, the coacervate was frozen overnight at −70 °C then freeze-dried for storage purposes (Labconco, Kansas city, MO, United States). The freeze-dried microcapsules were stored in airtight glass bottles, protected from light and kept in a desiccator until further studies were conducted [38].

#### 2.2.2. HPLC Method

The HPLC method was adapted from Ang et al. The HPLC analysis was performed using a Shimadzu-LC system (Shimadzu, Nakagyo-ku, Kyoto, Japan) equipped with a CBM-20A controller, LC-20AT pump, DGU-20A5 prominence degasser, SIL-20A auto-sampler, SPD-20AV detector and a CTO-10ASvp column oven. Chromatographic separations were achieved using the Thermo Hypersil Gold column (250 × 4.6 mm I.D.; 5 µm) guarded with C-18 guard column (Zorbax Eclipse Plus, Agilent, Santa Clara, CA, United States). The mobile phase which consists of acetonitrile and 2% acetic acid at the volume ratio of 40:60 (% *v*/*v*), was used with an isocratic elution at the flow rate of 1.3 mL/min. The column temperature was set at a standard of 35 °C with UV detection at 370 nm. The injection volume for each solution was 20 µL with a run time of 18.5 min. Data was acquired and processed using the LC-Solution Software (LC2010, Shimadzu, Nakagyo-ku, Kyoto, Japan). Solvents and distilled water were filtered via a 0.45 µm nylon membrane prior to being used in the HPLC method.

#### 2.2.3. Disruption of Microcapsules

The process of disruption of the microcapsules started by having the lyophilized microcapsules accurately weighed in a test tube. Next, 5 mL of 1% *w*/*w* acetic acid were added to the microcapsules. The mixture was then vortexed and sonicated at 40 °C for 30 min. After which, 5 mL of absolute ethanol was added and vortexed for another minute. The mixture was then incubated in a shaker bath (Memmert GmbH, Schwabach, Germany) and shaken overnight at a fixed temperature of 37 °C. The precipitate was separated by centrifugation at 4000 rpm for 10 min. The supernatant was then collected and filtered through a 0.45 µm nylon syringe filter. Lastly, the curcuminoids content was quantitated using the HPLC method.

#### 2.2.4. Microcapsule Characterizations

Microencapsulation efficiency: The entrapment efficiency, drug loading and encapsulation yield were calculated using the following equations:

$$\mathrm{Entrapment}\;\mathrm{efficiency}\;\left(\%\right)=\frac{\mathrm{Weight}\;\mathrm{of}\;\mathrm{drug}\;\mathrm{in}\;\mathrm{microcapsules}}{\mathrm{Weight}\;\mathrm{of}\;\mathrm{drug}\;\mathrm{fed}\;\mathrm{initially}}\times \;100$$

$$\mathrm{Drug}\;\mathrm{loading}\;\left(\%\right)=\frac{\mathrm{Weight}\;\mathrm{of}\;\mathrm{drug}\;\mathrm{in}\;\mathrm{microcapsules}}{\mathrm{Weight}\;\mathrm{of}\;\mathrm{microcapsules}}\times \;100$$

$$\mathrm{Encapsulation}\;\mathrm{yield}\;\left(\%\right)=\frac{\mathrm{Weight}\;\mathrm{of}\;\mathrm{microcapsules}}{\mathrm{Weight}\;\mathrm{of}\;\mathrm{polymer}\;\mathrm{and}\;\mathrm{drug}\;\mathrm{fed}\;\mathrm{initially}}\times 100$$

The amount of curcuminoids content was determined by using the HPLC method described in Section 2.2.2.

##### Optical Microscopy Observation

The morphology of moist microcapsules suspended in distilled water was observed directly using an optical microscope (Olympus BXDP72, Shinjuku, Tokyo, Japan) equipped with an imaging system (Olympus BX53, Shinjuku, Tokyo, Japan), aided by cellSens software for clear image acquisition.

##### Wall Thickness

The wall thickness of the microcapsules produced was also determined using an optical microscope (Olympus BXDP72, Shinjuku, Tokyo, Japan) equipped with the same imaging system (Olympus BX53, Japan), with cellSens software. The values of 150 particles were averaged.

##### Particle Size

The microcapsule particle size was determined by utilizing the laser diffraction system (Mastersizer S-Long Bed Laser Analyzer, Malvern Instruments, Malvern, Worcestershire, United Kingdom) fitted with a small sample dispersion unit (MS1) connected to a dispersion unit controller. The results were expressed in volume mean diameter, D(4,3),size distribution, span (D(v, 0.9) − D(v, 0.1)]/D(v, 0.5)) and provision in the software of Mastersizer S. The analysis was carried out in triplicate manner to obtain an average.

##### Surface Characterization by Scanning Electron Microscope

The morphology of dried microcapsules was studied using an electron scanning microscope (LeoSupra 50 VP Field Mission SEM, Carl-Ziess SMT, Oberkochen, Baden-Wurttemberg, Germany). The Oxford INCA 400 energy dispersive X-ray microanalysis system (EDAX Inc., McKee Drive Mahwah, NJ, United States) software was applied for analyzing of the data. The samples were fixed on brass stubs using double-sided adhesive tape and then coated with gold under vacuum pressure.

##### Appearance and Stain Testing

The appearance of free curcuminoids and cross-linked curcuminoids microcapsules (CPM) dried powders were macroscopically examined to allow for clearer identification and differentiation of the two. The effect of color staining was tested by placing the respective samples on a dry and glossy paper, and then removing. If the sample adhered to the glossy paper, it was considered stained.

##### Differential Scanning Calorimetry (DSC)

The physical state of free curcuminoids and curcuminoids microcapsules (cross-linkedand non-cross-linked) powders were analyzed with the differential scanning calorimeter (Pyris 6, Perkin-Elmer, Waltham, MA, United States). A total of 5mg of each sample was accurately weighed using a microbalance (ME 5, Sartorius, Gottingen, Germany) in an aluminum pan and covered. The thermo grams of the samples were obtained at a scanning rate of 10 °C/min conducted over a temperature range of 0 to 450 °C under N_2_ parches. An empty, loosely covered, aluminum pan was used as a reference. The analysis of each sample was done in triplicate.

##### Fourier Transforms Infrared Spectroscopy

The intermolecular interactions of curcuminoids and coating materials (gelatin and chitosan) were investigated by FTIR analysis (Nexus FTIR, Thermo Nicolet Instrument Corporation, Madison, WI, United States). Prior to measurement, samples and potassium bromide (KBr) were both incubated at 40 °C in an oven for 2 h to remove moisture. KBr disk of the samples were prepared by mixing KBr with the material in the ratio of 100:1 % *w*/*w*, triturated in an agate mortar with pestle, then loaded into an evacuable potassium bromide die and compressed for 30 s by applying an IR hydraulic press (Hydraulic Unit Model #3192, Carver Laboratory Equipment, Wabash, IN, USA) of 9 tons to obtain a thin disk. FTIR spectrum of free curcuminoids, curcuminoids microcapsules and drug-free microcapsules were scanned with a deuterated tri-glycine sulfate (DTGS) detector from 4000 to 500 cm^−1^. For each spectrum, 32 consecutive scans at 4 cm^−1^ resolution were averaged. OMNIC software was applied for analyzing the data.

#### 2.2.5. Swelling Studies

The swelling studies method was adapted from Lin et al. with a few modifications to fit the current study [39]. A total of 100mg microcapsules was weighed in a glass tube, where 10 mL of medium was then added; either one of the mediums were used: (a) 0.1 M citrate-phosphate pH 5.4, (b) 0.01 M phosphate buffer saline pH 7.4 or (c) 95% ethanol. The tube was then capped and placed in a water bath shaker (Memmert GmbH, Schwabach, Germany) for 4 h at 35°C. The microcapsules were collected and blotted with Whatman filter paper no. 1 to remove any moisture on the surface and then weighed. The swelling percent of microcapsules was calculated using the following formula:

$$\mathrm{Swelling}\;\left(\%\right)=\;\frac{W_{t}-W_{0}}{W_{0}}\times \;100$$

where W_t_ represents the weight of the microcapsules at the 4th h in the medium and W_0_ is the initial weight of the dry microcapsules.

#### 2.2.6. Permeation Study by Using Franz Diffusion Cell

An in vitro drug permeability study was carried out using standard 9 mm jacketed clear glass static Franz cells (PermeGear, Hellertwon, PA, United States) with the receptor volume of 5 mL. Cellulose nitrate membrane (13 mm diameter with pore size 0.45 µm, Whatman GmbH, Hahnestraße, Dassel, Germany) was used as a synthetic membrane for the permeation experiments. Citrate-phosphate buffer with pH 5.4 containing 0.5% *w*/*w* Tween 80 was filled into the Franz diffusion cell through the sampling port aided by a syringe which was mounted with a 10-cm long, thin tubing. The cellulose nitrate membrane was ensured to be in contact with the fluid and no bubble was formed below the membrane. Three sets of Franz cells were placed on #V3 3-Station Franz Cell Stirrer (PermeGear, Hellertwon, PA, United States) which served for temperature circulation and provided a constant stirring rate of 500 rpm. The apparatus was kept in the Franz cells at a constant temperature of 37 ± 0.5 °C due to the circulating water of an external water jacket. After 15 min of equilibration of the membrane with the receptor solution, 150 µg of free curcuminoids or 960 µg curcuminoids microcapsules (equivalent to 150 µg curcuminoids content) were accurately placed on the surface membrane of the donor compartment for separate analysis. Receptor solution of 200 µL aliquots were withdrawn at various time intervals (i.e., 0.25, 0.5, 1, 2, 4, 6, 8, 10, 12, 24, 36, 48 and 72 h) through the sampling port by using a 1-mL disposable syringe mounted with a 10-cm long, thin tube. The cells were refilled with fresh solution to keep the volume of the receptor solution constant throughout the experiment. All openings, including the donor top and receptor port, were occluded with parafilms to prevent evaporation. The experimental setup was protected from all incidence of light. All experiments for each sample were conducted in triplicate. The curcumin content in the receptor solution was quantified by the HPLC method. The amount of drug released was plotted versus time.

#### 2.2.7. Drug Release Kinetics Study

The drug release kinetics and mechanisms of free curcuminoids and microcapsules were examined by fitting the drug release data to the following mathematical model [40,41,42]:

Zero order kinetics: Q_t_ = Q_0_ + K_0_t (1)

where Q_t_ is the percent of drug released at time t, Q_0_ is the initial amount of drug in the solution (most times, Q_0_ = 0), K_0_ is the zero order release constant expressed in units of concentration/time and t is the time in hours. The data obtained from in vitro drug release studies were plotted as percent drug released versus time.

First order kinetics: log Q_t_ = log Q_0_ + K_1_t (2)

where Q_t_ is the amount of drug released in time t, Q_0_ is the initial concentration of drug and K_1_ is the first order release constant expressed in unit of time^−1^. The data obtained from in vitro drug release studies were plotted as log percent of drug remaining (log Q_0_ − Q_t_) versus time which will be linear if first order obeys with a negative slope, −K_1_/2.303.

Higuchi model: Q_t_ = K_H_(t)^1/2^ (3)

where Q_t_ is the amount of drug released at time t per unit area and K_H_ is the Higuchi dissolution constant. The data obtained from in vitro drug release studies were plotted as percent drug release versus square root of time.

Hixson–Crowell model: W_0_^1/3^ − W_t_^1/3^ = K_s_t (4)

where W_t_ is the remaining amount of drug in the dosage form at time t, W_0_ is the initial amount of the drug in pharmaceutical dosage form and K_s_ is the rate constant for the Hixson–Crowell rate equation. In this model the cube root of percent drug remaining in the matrix (W_0_^1/3^ − W_t_^1/3^) versus time is linear.

Korsmeyer–Peppas model: M_t_/M_∞_ = Kt^n^ or log (M_t_/M_∞_) = log K + n log t (logarithm form) (5)

where M_t_/M_∞_ is the fraction of released drug at time t, n is the release exponent indicative of the mechanism of transport of drug through the polymer, K is the kinetic constant (having units of t^−n^) incorporating structural and geometric characteristics of the delivery system. Data obtained from in vitro drug release studies were plotted as log percent drug released versus log time.

The mechanism of drug release was indicated by the Korsmeyer–Peppas model [43] where the first 60% drug release data (M_t_/M_∞_ < 0.6) were fitted in the curve. The value of n was used to characterize the release mechanism of the drugs [44].

#### 2.2.8. Light Stability

Free curcuminoids and cross-linked curcuminoids microcapsules dried powders were weighed to be exactly 400 mg and placed in an enclosed glass petri dish (diameter of 9 cm) each. The samples in the petri dish were less than 2 mm thick and were kept in a desiccator at room temperature (28 ± 4 °C/75 ± 10% RH), allowing them to receive sunlight exposure for one month. The drug content was then analyzed using the HPLC method after exposure to sunlight for 0, 5, 10, 15, 20, 25 and 30 days. All measurements were performed in triplicate. The results were reported in percent drug content using the following formula where mean value was recorded:

$$\mathrm{Drug}\;\mathrm{content}\;\left(\%\right)=\;\frac{\mathrm{drug}\;\mathrm{content}\;\mathrm{at}\;\mathrm{time}\;\mathrm{point}\;t}{\mathrm{drug}\;\mathrm{content}\;\mathrm{at}\;\mathrm{time}\;\mathrm{point}\;0\;}\times \;100$$

#### 2.2.9. Storage Stability

Free curcuminoids and cross-linked curcuminoids microcapsules dried powders were stored in three conditions for long-term (12 months) and accelerated (6 months) stability studies (triplicate preparations for each condition): (1) kept in desiccator at room temperature (28 ± 4 °C/75 ± 10% RH) for 12 months, (2) kept in refrigerator (5 ± 3 °C) for 6 months, and (3) kept in humidity chamber (40 ± 2 °C/75 ± 5% RH) for 6 months; all samples were protected from light. Curcumin content was analyzed using HPLC and particle size was measured by utilizing the laser diffraction system (Mastersizer S-Long Bed, Malvern Instruments, Malvern, Worcestershire, United Kingdom) at 0, 3 and 6 months for samples stored at accelerated stability conditions (40 ± 2 °C and 5 ± 3 °C). Samples stored at room temperature (28 ± 4 °C) were analyzed at 0, 3, 6 and 12 months. At each time interval, 10 mg of free curcuminoids were dissolved using 100 mL absolute ethanol, whereas 20 mg of microencapsulated curcuminoids were first disrupted and then diluted in 100 mL absolute ethanol for drug content quantification. All measurements were performed in triplicate. Percentage of drug (curcumin) content was reported in mean value. The drug content of each 0-month samples were used as the control.

#### 2.2.10. Determination of Residual Formaldehyde by Gas Chromatography (GC)

Analysis of formaldehyde was performed with an Agilent flame ionization detector (FID) (Agilent, Santa Clara, CA, United States) equipped with the Agilent GC 6890 series (Agilent, USA). The column used was an Agilent HP5 fused silica capillary column (30 m × 0.32 mm, film thickness 0.32 µm). The injector and detector temperatures were 200 °C and 250 °C, respectively. Hydrogen (7.39 psi on column) was the chosen carrier gas. The initial oven temperature was set at 40 °C, held for 3 min and increased to 130 °C after ramping at 20 °C/min. The total run time for the analysis was 7.5 min.

The 20 mL headspace vials were incubated in the headspace of an auto-sampler (Agilent G1888 headspace sampler) at 90 °C for 15 min. After equilibration, 0.2 mL of the headspace vial was pressurized into GC/FID. The loop and transfer line temperatures were set at 95 °C and 100 °C, respectively.

## 3. Results

### 3.1. Microencapsulation Efficiency, Morphology, Wall Thickness and Particle Size

The results of entrapment efficiency, drug loading, entrapment yield, particle size and capsule wall thickness of curcuminoids microcapsules are tabulated in Table 1.

**Figure 1:**
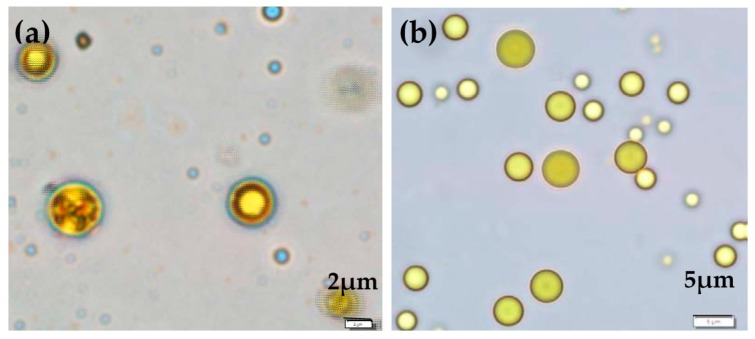
Micrographs show the configuration of (**a**) cross-linked curcuminoids microcapsules (CPM) and (**b**) non-cross-linked CPM.

The morphology of moist microcapsules suspended in distilled water was observed under an optical microscope with the aid of an image analyzer. The microcapsules were spherical and enveloped by a shell wall (Figure 1).

### 3.2. Surface Characterization by SEM

**Figure 2:**
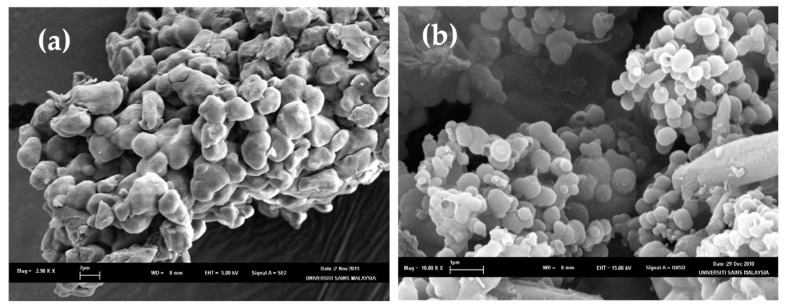
Scanning electron microscopes (SEMs) for (**a**) non-cross-linked CPM (scale: 2.98 K) and (**b**) cross-linked CPM (scale: 10 K).

The SEM images of cross-linked and non-cross-linked curcuminoids microcapsules are shown in Figure 2. The lyophilized cross-linked CPM (Figure 2a) maintained the wall integrity and the agglomerated particles were spherical and had a smooth surface. On the contrary, the configurations of the non-cross-linked CPM were irregular, and the agglomerated particles were coalesced (Figure 2b).

### 3.3. Appearance and Staining Testing

**Figure 3:**
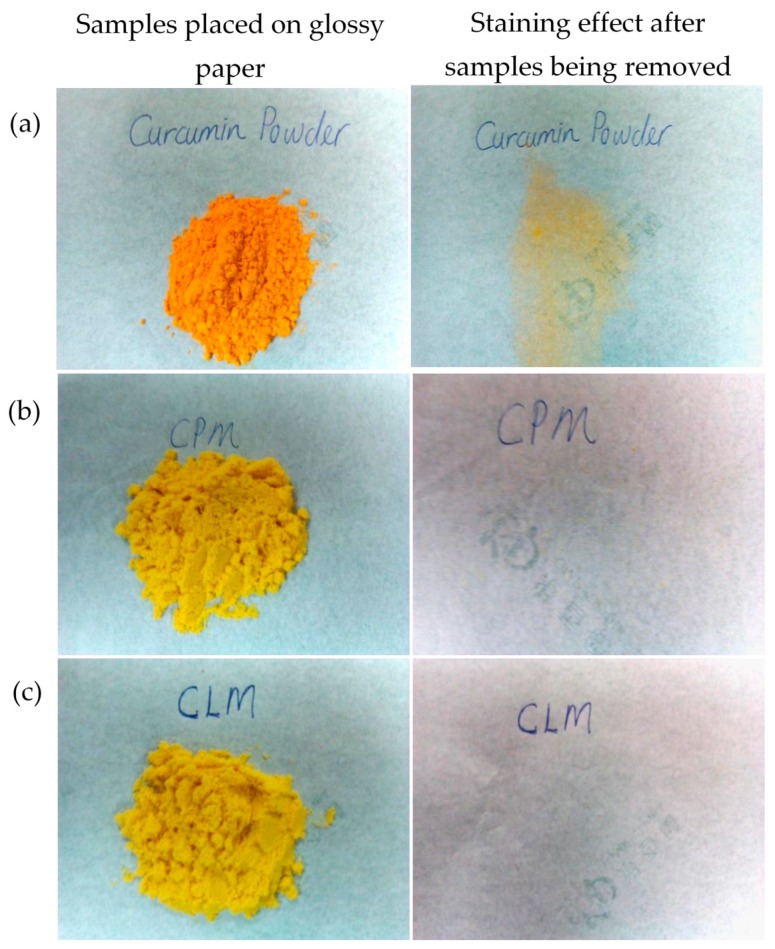
Pictures show the physical appearance and the staining effect of: (**a**) native curcuminoids powder, (**b**) cross-linked CPM and (**c**) non-cross-linked CPM. * CLM: non-crossed-linked.

The photos of free curcuminoids (non-encapsulated), cross-linked CPM and non-cross-linked CPM powders are shown in Figure 3. It can be seen that there are differences in the colors of the free (non-encapsulated) curcuminoids and the microencapsulated curcuminoids. The free curcuminoids portray a bright orange color whereas the CPM appear to be yellow in color. The staining test on a glossy paper shows that the free curcuminoids were sticky whereas the cross-linked CPM and non-cross-linked CPM did not leave any sort of stain after being removed from the paper. Moreover, the microcapsules were free flowing compared to the free drug powders.

### 3.4. Differential Scanning Calorimetry (DSC)

**Figure 4:**
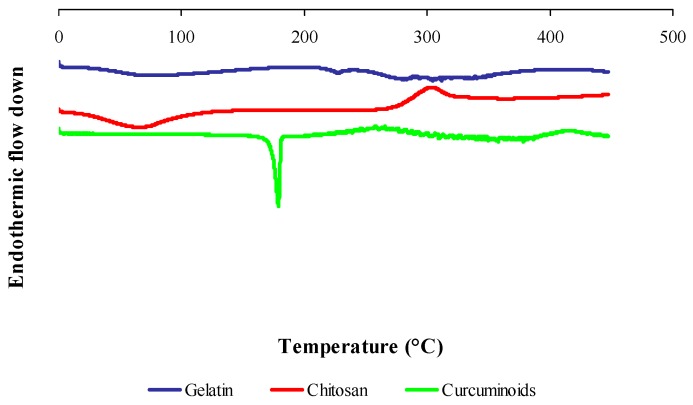
Differential scanning calorimetry (DSC) thermograms of raw material powders: gelatin, chitosan and curcuminoids.

Figure 4 shows the DSC thermogram of gelatin, chitosan and curcuminoids powders. Five endothermic peaks were observed in the gelatin thermogram. A broad endothermic peak at 74.20 °C was attributed by the denaturation of the gelatin segments, which was related to the helix-coil transition of collagen [45]. The endothermic peaks at 227.60 °C, 279.76 °C and 288.77 °C were due to thermal degradation, and the peak at 304.10 °C was attributed to the thermal breaking of peptide bonds in the gelatin chain [46]. The DSC thermogram for chitosan shows an endothermic peak at 66.69 °C contributed by the loss of water, and an exothermic peak at 303.59 °C corresponding to the decomposition of amine (GlcN) unit [47]. The endotherm at 179.02 °C in the free curcuminoids thermogram was due to the curcumin melting point and an exothermic peak of 204 °C was corresponding to its gradual decomposition [48].

**Figure 5:**
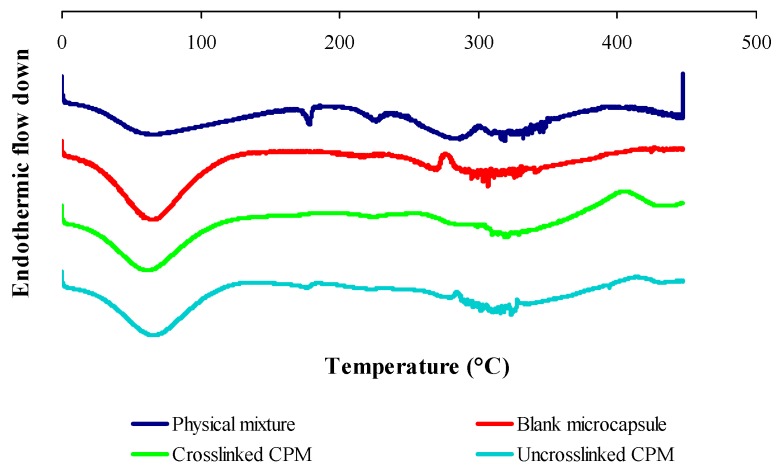
DSC thermograms of physical mixtures of raw materials, blank microcapsules, cross-linked and non-cross-linked curcuminoids microcapsules (CPM).

Figure 5 shows the DSC thermogram of the physical mixture of raw materials, blank microcapsule (drug-free microcapsule), cross-linked and non-cross-linked curcuminoids microcapsules (CPM). The physical mixture of raw materials shows all the feature peaks as seen in an individual thermogram presented in Figure 4. The blank microcapsule demonstrated a broad endothermic peak centered at 67.16 °C attributed to free water removal from the system and an exothermic peak at 303.38 °C due to the thermal breakdown of the peptide bonds from the gelatin-chitosan complex. The cross-linked CPM showed a denaturation enthalpy at 61.34 °C while the disappearances of an exothermic peak of chitosan and some thermal peaks of gelatin in the DSC thermogram of the cross-linked CPM reveal the existence of covalent bonds. The disappearance of some thermal features of the biomaterials in the microcapsule suggest that there were chemical interactions. The DSC curve for the non-cross-linked CPM shows the presence of mainly thermal peaks of drug and polymers, which changed the enthalpy of fusion. The endothermic peaks which corresponded to the melting point of curcumin shifted to 176.10 °C; the endothermic peak of gelatin which was attributed by the thermal breaking of peptide bonds in the gelatin chain [46] shifted to 318.46 °C; and the exothermic peak of chitosan shifted to 284.29 °C. The existence of a definitive melting point of curcumin indicated that the drug was dispersed in solid state in the core of a non-cross-linked CPM microcapsule.

DSC scans also revealed that microencapsulation extended the decomposition temperature of encapsulated curcuminoids, where the cross-linked CPM remained stable up to 400 °C and the non-cross-linked CPM only showed stability up to 300 °C. These results were comparable to the free curcuminoids which started to decompose at 204 °C under oxidative conditions. These results were suggestive of the enhanced thermal stability of curcuminoids due to microencapsulation.

### 3.5. Fourier Transforms Infrared Spectroscopy

**Figure 6:**
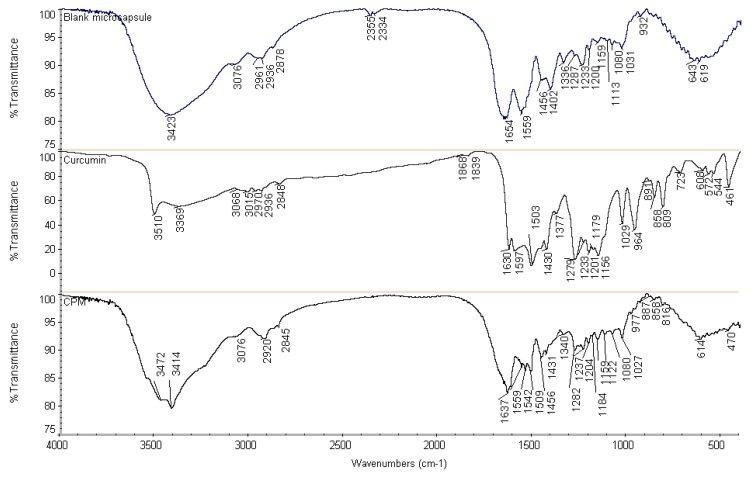
Fourier transform infrared (FTIR) spectra of blank microcapsule, free curcuminoids and cross-linked CPM.

In FTIR scanning, the free curcuminoids spectrum (Figure 6) portrays characteristic phenolic O–H stretching vibration bands at 3510 cm^−1^ and 3369 cm^−1^, C=C stretching vibration band at 1630 cm^−1^ and aromatic C=C stretching vibration band at 1597 cm^−1^. Multiple bands were observed in the range of 3000–2800 cm^−1^ which can be attributed to the symmetric and asymmetric aromatic C–H stretching vibrations. The absorption peak at 1503 cm^−1^ was identified to the mixed C=O and C=C stretching vibrations, and the peak at 1458 cm^−1^ was assigned to the C=C stretching vibration of benzene rings. The bands at 1430 cm^−1^ and 1377 cm^−1^ were attributed to C–H stretching and deformation of methyl groups, respectively. On the other hand, phenolic C–O stretching vibrations were responsible for the development of absorption bands at 1279 cm^−1^; the C–O–C stretching vibration band at 1029 cm^−1^; and the aromatic C–H out-of-plane bending vibration band at 964 cm^−1^ [49].

FTIR analysis of cross-linked CPM (Figure 6) was evaluated with all characteristic absorption peaks of curcuminoids observed on the CPM spectrum as well. However, the spectrum showed a decrease in amplitudes of amide I and amide II bands, in comparison with a blank microcapsule. The vibration band at 1654 cm^−1^ in the spectrum of the blank microcapsule demonstrated a shift to 1637 cm^−1^ in the CPM spectrum. This might be due to the interaction involving –C=O and –NH groups, which could be intra, intermolecular or both. Meanwhile, small shoulder peaks were observed at the peaks of amide I and amide II in the CPM spectrum, which were due to overlapping of the dominant stretching and bending vibration bands of polymers to the absorption bands of curcuminoids. The characteristic peaks were found in the CPM spectrum, confirming curcuminoids present in the dispersed condition. In addition, cross-linking with formaldehyde could be the contributing factors towards the decrease in intensity and frequency of amide I and amide II bands. For instance, the methylene glycol (HO-CH_2_-OH, formaldehyde in water) will potentially react with its neighboring nitrogen atom to form a methylene bridge (–CH_2_–) by releasing H_2_O. Thus, the strength of C–N stretching and N–H bending will show an increased level, subsequently lowering the frequency and amplitude of these vibration bands. The peaks at 1542 cm^−1^ and 1538 cm^−1^ representing N–CH_2_ bond was evident towards the formation of covalent linkages in the gel network. The nitrogen atom from amino acid and the nitrogen atom from peptide bond (–NH–CO–) were cross-linked with formaldehyde in the formation of a methylene bridge (–NH-CH_2_-NH–). The fixations of formaldehyde led to an intramolecular and intermolecular cross-linking network. On the other hand, C=O stretching vibration of the aldehyde group was not found at the absorption peak of 1730 cm^−1^. Hence, it could be concluded that no free formaldehyde was attached on the surface of the microcapsules, thus, the formulations are considered safe for topical usage. The disappearance of absorption bands at 1597 and 964 cm^−1^ indicate the inclusion of curcuminoids inside the core of said microcapsule.

### 3.6. Swelling Studies

**Figure 7:**
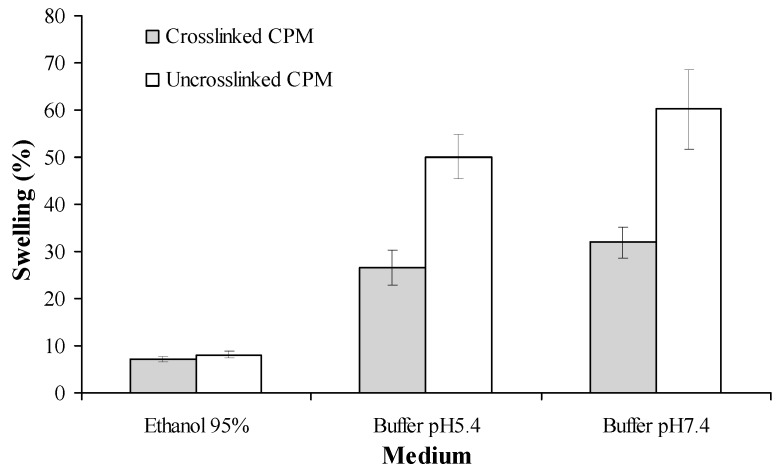
Swelling indexes of curcuminoids microcapsules in different media at 35 ± 2 °C. Mean ± standard deviation (SD), *n* = 3.

The swelling indexes of cross-linked and non-cross-linked CPM in different media were observed at the fourth h of swelling. As shown in Figure 7, the highest swelling index was seen in the phosphate buffer saline with pH 7.4, while the lowest was in 95% ethanol. The water absorption capacity of cross-linked CPM was significantly lower as compared to the non-cross-linked version.

### 3.7. Permeation Studies by Franz Diffusion Cell

**Figure 8:**
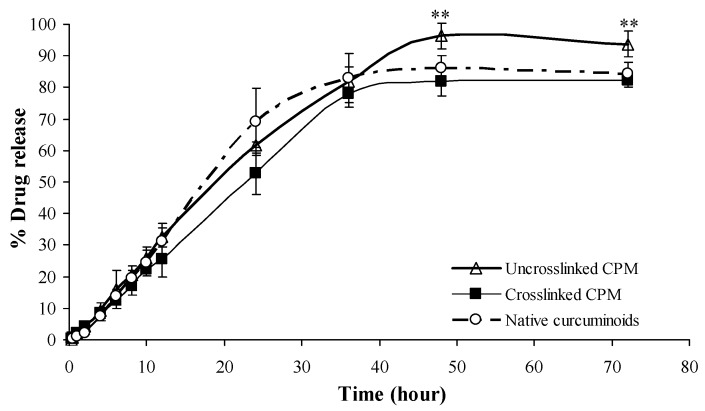
Release profiles of free curcuminoids, cross-linked and non-cross-linked curcuminoids microcapsules (CPM). Mean ± SD, *n* = 3. ** indicates *p* < 0.01 as compared with native curcuminoids group.

The drug release profiles of free (non-encapsulated) curcuminoids, cross-linked CPM and non-cross-linked CPM are shown in Figure 8. Microencapsulation without cross-linking enhances the solubility of curcuminoids. On the other hand, cross-linked CPM retards the drug released rate from its microcapsule. At the end of the test period (72 h), the total drug content in the solution was 96%, 86% and 81% for non-cross-linked CPM, free curcuminoids and cross-linked CPM, respectively.

### 3.8. Permeation Studies by Franz Diffusion Cell

The drug release profiles of free (non-encapsulated) curcuminoids, cross-linked CPM and non-cross-linked CPM are shown in Figure 8. Microencapsulation without cross-linking enhances the solubility of curcuminoids. On the other hand, cross-linked CPM retards the drug released rate from its microcapsule. At the end of the test period (72 h), the total drug content in the solution was 96%, 86% and 81% for non-cross-linked CPM, free curcuminoids and cross-linked CPM, respectively.

### 3.9. Drug Release Kinetics Study

The coefficient of determination (R^2^) and rate constants (K) of different kinetic models are tabulated in Table 2. The free curcuminoids and microencapsulated curcuminoids both follow the zero order kinetics. The cross-linked CPM showed the longest duration of drug release with t_50%_ of 22.75 h in comparison with the non-cross-linked version of 19.02 h followed by free curcuminoids, which took 17.18 h. In addition, the release mechanisms of all samples were characterized as super case-II transport (erosion of the polymeric chain) because their n values were higher than 0.85 [50].

### 3.10. Light Stability

The light stability of free curcuminoids and cross-linked CPM were studied by exposing the samples to daylight for 30 days at room temperature (28 ± 4°C/75 ± 10% RH). The simple order reaction models such as zero-, first-, and second order kinetics were used to describe and determine the kinetics of drug degradation for each sample. It was found that the photo degradation of free curcuminoids and cross-linked CPM followed zero order kinetics based on the best fitted determination of coefficient (R^2^) values (Table 3). The rate constants for the samples were obtained from their respective best fitted kinetic plots and were used to calculate the half-life degradation (t_1/2_) of the studied samples. The cross-linked CPM showed extended t_1/2_ up to 236.74 days compared to free curcuminoids which had t_1/2_ of 66.63 days. 

### 3.11. Storage Stability

**Figure 9:**
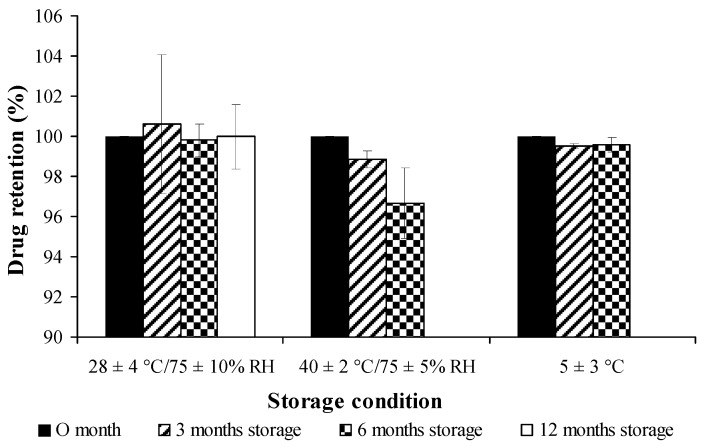
Stability study of free curcuminoids dried powder on percent drug retention at different conditions.

**Figure 10:**
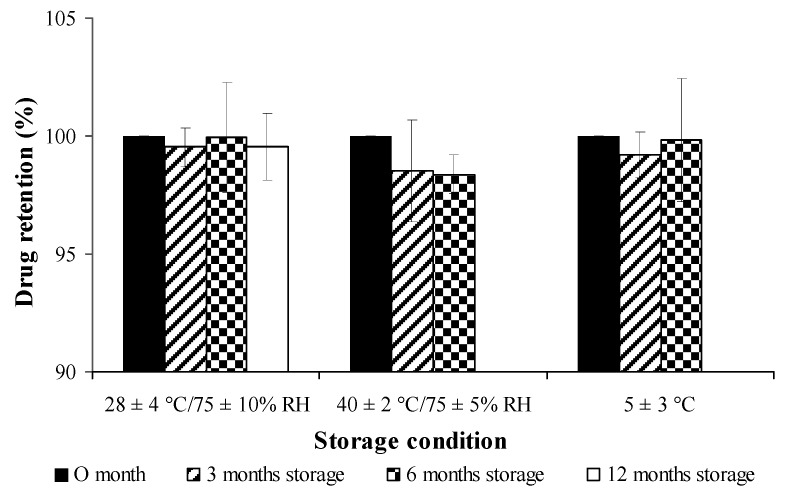
Stability study of cross-linked curcuminoids microcapsule lyophilized powder on percent drug retention at different conditions.

**Figure 11:**
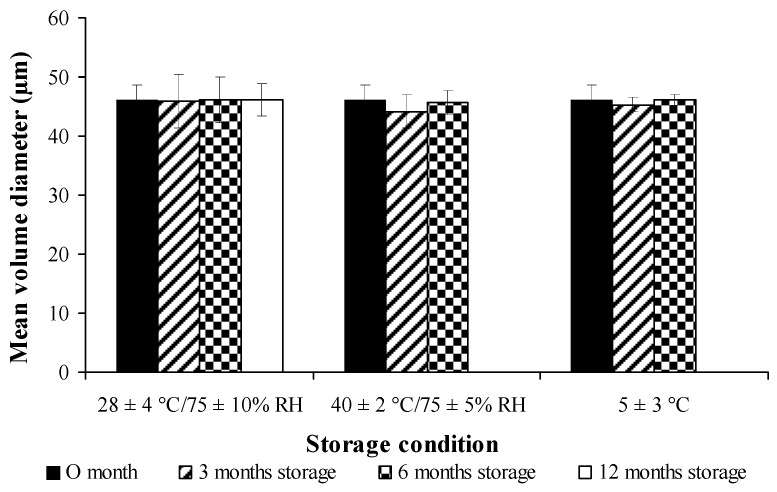
Stability studies of cross-linked curcuminoids microcapsule lyophilized powder on particle size at different conditions.

The percentage of drug retention for both free curcuminoids and cross-linked CPM in long-term storage condition (28 ± 4 °C /75 ± 10% RH for 12 months) and accelerated storage conditions (40 ± 2 °C/75 ± 5% RH and 5 ± 3 °C, for 6 months) are shown in Figure 9 and Figure 10, respectively. Both free and encapsulated curcuminoids showed excellent chemical stability during storage periods. In addition, the microcapsule was stable and had no significant changes in particle size during storage (Figure 11).

On the other hand, the sensorial characteristics of the samples were not significantly different from their original properties, and the microcapsule samples remained as orange fine powders throughout the duration of the study.

### 3.12. Determination of Residual Formaldehyde by Gas Chromatography

**Figure 12:**
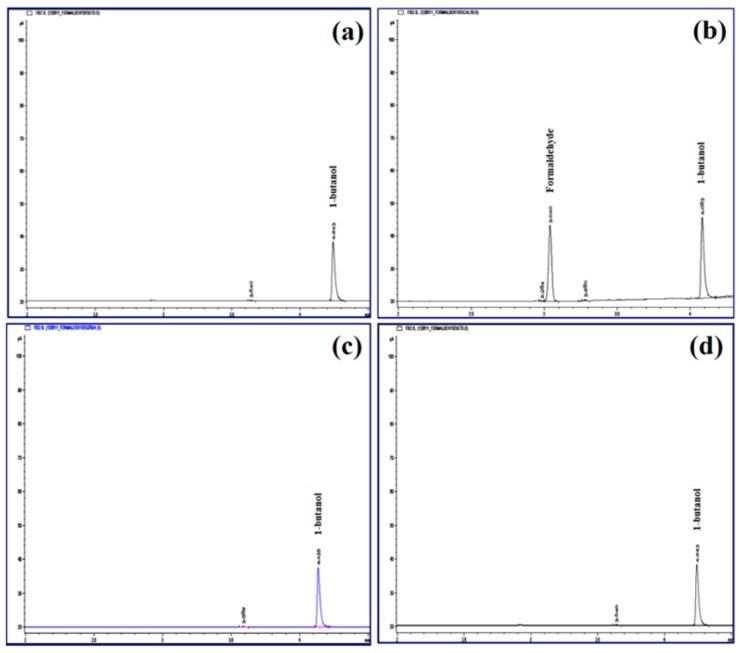
Chromatograms of formaldehyde analysis by gas chromatography: (**a**) blank solution, (**b**) internal standard solution of 1-butanol, (**c**) standard solution containing 0.1% formaldehyde and 1-butanol, and (**d**) cross-linked CPM.

The residue of formaldehyde in lyophilized cross-linked CPM was determined by using gas chromatography. Internal standard of 1-butanol was used as the reference peak. The retention time for formaldehyde and 1-butanol was 3.05 and 4.05 min, respectively. The trace residue of formaldehyde in cross-linked CPM was analyzed, and there was no formaldehyde detected (Figure 12).

## 4. Discussion

Microencapsulation of curcuminoids with or without cross-linking did not demonstrate significant differences in terms of entrapment efficiency and drug loading. Curcuminoids microcapsules with or without cross-linkages were surrounded by a layer of coacervate wall with curcuminoids encapsulated in the core. However, cross-linked CPM had a thicker coacervate wall and a smaller mean volume diameter as compared to non-cross-linked CPM. This could be due to the fact that cross-linking conferred a particular rigidity to the coacervate wall. Formaldehyde can react with the nitrogen atoms on amide and amine groups of gelatin and chitosan to form covalent bonding, hence strengthening the capsule gel network. Additionally, covalent cross-linking reduces the swelling degree of microcapsules during dispersion in distilled water. On the other hand, cross-linking was shown to be able to protect the capsule wall integrity during the freeze-drying process, thus allowing the maintenance of integrity for the cross-linked microcapsules. In addition, the compact ionic bond and covalent cross-links results in the smooth and nonporous surface of the particles. Freeze-drying also resulted in the production of solid fine powder of microcapsules which were not sticky, non-staining and free flowing.

DSC was used to study the physical state of the drug in microcapsules by examining the changes of melting point and shape on the DSC thermogram. Persistence of endothermic peak and exothermic peak of the mixture constituents indicates no significant interaction between the materials. The blank microcapsules and the curcuminoids microcapsules showed a decrease of denaturation enthalpy. This phenomenon could be due to an increase in covalent bonds with a decrease of hydrogen bonding. In general, complex coacervation is an electrostatic driven interaction, where the interactions of the negatively-charged gelatin and the positively-charged chitosan form peptide bonds between the ammonium and carboxylate groups. Thereby, the hydrogen bonds within molecules involving hydroxyl, carboxylate, and amine or ammonium groups will be reduced due to unavailability of functional groups. In addition, cross-links induce covalent linkage within and between molecules, thus possibly reducing the number of hydrogen bonds. The intra and intermolecular interactions and the formation of bonds explain the change of enthalpy. The breaking of hydrogen bonds is an endothermic fusion, while the breaking of covalent linkages is an exothermic reaction [51]. The curcuminoids in the core of the microcapsule are in a crystalline state and microencapsulation leads to an enhancement of the heat and oxidative stability of curcuminoids by increasing the compound decomposition temperature, and cross-linked microencapsulated curcuminoids showed an advanced heat stability in comparison to the non-cross-linked version.

FTIR is a mainstay among techniques used for determination of the molecular structure of proteins (e.g., gelatin), polysaccharides (e.g., chitosan) and drugs (e.g., curcuminoids). FTIR analysis therefore will be able to confirm the compatibility of gelatin and chitosan in the formation of complex coacervation via intermolecular interactions between the –NH_2_ and –COO^–^ groups. Meanwhile, it is postulated that the curcuminoids entrapped in the core of the microcapsule maintained their chemical structure. The hydroxyl (–OH), amine (–NH_2_) and carboxylate (COO^–^) groups of gelatin are capable of forming hydrogen and/or peptide bonds within the molecules or between molecules with the hydroxyl (–OH) and ammonium (–NH_3_^+^) groups of chitosan during formation of the complex [52]. As a consequence of these interactions, the amplitudes and the intensities of the absorption bands of those groups were affected. Formaldehyde used in cross-links formed methylene bridges which strengthened the gel network of microcapsules; more or less changing the amplitude and the intensity of the absorption bands of peptide groups. On the other hand, FTIR revealed that no residue formaldehyde was found in the microcapsules.

In aqueous solution, microcapsules would interact with the H_2_O molecules through their available –COO^−^ and –NH_3_^+^ groups of polymers, resulting in the swelling of the microcapsules. Cross-linking of the microcapsules were shown to lower the swelling degree of microcapsules, with similar findings being reported by other authors [51,53]. This is because the formation of cross-linkage between the aldehyde groups of formaldehyde and amino groups of coating materials reduces the number of free amino groups on chitosan or gelatin, hence reducing the availability of these amino groups to interact with H_2_O molecules in the medium. In addition, due to the tight structure of gelatin-chitosan, there was a reduction in the exposure of ionic groups making the cross-linked microcapsules more lipophilic in capsule walls [13,54].

In vitro drug release studies were performed using citrate-phosphate buffer pH 5.4 containing 0.5% *w*/*w* Tween 80 as the dissolution medium to evaluate the feasibility of coacervation microencapsulation for controlled release of drugs in topical application. Prior to the experiment, solubility of the curcuminoids in the medium was quantified using the HPLC method. The amount of sample loaded on the membrane was less than 30% of the maximum solubility to ensure the curcuminoids could be dissolved completely and sink condition was maintained during the entire release study. Microencapsulation shows a positive effect in drug solubility. On the other hand, cross-linked microcapsules demonstrated prolonged duration of drug release due to the formation of intermolecular covalent linkages in the gel network.

Zero order kinetics is the ideal kinetic profile to describe the release of a poorly soluble drug where the matrix does not disaggregate. This type of release profile is independent of the constant concentration of drugs, whereby the drugs are being released from the matrix over a specific period of time to achieve a prolonged pharmacological action, hence providing a steady therapeutic drug level to the human body over a long period of time [40,50,55,56].

The half-life of drug release (t_50%_) is the time required for 50% of a drug to be released from the dosage form which is the best in vitro variable in correlation with the in vitro activity [57]. The rate of drug release from non-cross-linked microcapsules was shown to be faster than cross-linked microcapsules possibly due to the looser network of polymeric walls of non-cross-linked microcapsules, and the porous structure of capsule walls as compared to the reticulated polymer network found in cross-linked microcapsules. Therefore, swelling and erosion of non-cross-linked microcapsules causes faster release of a drug. The higher degree of polymer relaxation of the non-cross-linked microcapsules is indicated by the higher value of n (Korsmeyer–Peppas’ release exponent). In the fundamentals of cross-linked microcapsules, the hydrogen, peptide bonds and aldehyde cross-links compete with each other actively. These interactions can take place selectively or simultaneously leading to a strong polymeric wall that is capable of retarding the ingress of water; thus, the embedded drug would slowly dissolve and be released at a constant rate over a longer period, increasing the half-life of release of the drugs. In this study, cross-linked CPM presented a lower swelling capacity and drug release rates compared to the non-cross-linked microcapsules. The findings of this study were synonymous to the findings reported by Alvim and Grosso [53].

Cross-linked and non-cross-linked CPM followed the super case-II transport mechanism. This comes to show that drug release mechanisms were due to both diffusion and relaxation of the polymer chain [41,58,59] at different degrees depending on the swelling capacity of these microcapsules.

As commonly recognized, polyphenols are sensitive to light, heat, oxygen and can easily deteriorate when being exposed to these environmental factors [60,61]. Light in the form of energy can initiate and accelerate decomposition [31], and while many attempts have been made to stabilize these photo-labile drugs, microspheres and microcapsules have shown to have the capability to impart a high degree of protection from light and demonstrated both enhanced quality and stability [31]. The drug degradation of cross-linked CPM follows the zero order reaction kinetics indicating that the rate of degradation was time-dependent. Half-life is the time required for a drug to decompose to one-half of the original concentration [62], thus the extended half-live of the microcapsule indicates that microencapsulation enhanced photo-stability of said curcuminoids.

The cross-linked CPM demonstrated negligible reduction of drug content and growing of particle size throughout the stability test. The stability may be contributed by protection of the strong polymeric wall of electrostatic interactions and covalent cross-linkages. In the gas chromatography analysis, the results shown were coincidently similar to FTIR analyses at which there has no formaldehyde been detected in the microcapsule. Therefore, it can be safely concluded that the microcapsules are safe for topical application.

## 5. Summary

Curcuminoid microcapsules are spherical structures with definitive characteristics of being discrete, free flowing and having a reduced color staining effect. One of the major upsides in development of the microcapsules for curcuminoids are the integrity and stability of the drugs which are superior to free curcuminoids due to the thick, polymeric walls of the capsules. Microencapsulation in general leads to a higher heat, light and oxidative resistance for the curcuminoids in the core which results in lower degradation rates of the drugs. Enhancement towards the microencapsulated version was shown by the cross-linkages formed in the capsules. The cross-linkages increase the density of the polymeric capsule walls, not only further elevating the level of resistance against degradation of the drug, but also increasing the solubility of the drugs. All in all, these microcapsules demonstrated an ideal zero order release kinetics with a super case-II transport mechanism that fundamentally suggest that these curcuminoid microcapsules can be incorporated towards a topical delivery system for the sustained release option of drugs.

## Figures and Tables

**Table 1:** Characterizations of curcuminoids microcapsules (CPM).

Parameters	Cross-Linked CPM	Non-cross-Linked CPM
Entrapment efficiency (%)	82.69 ± 0.93	81.92 ± 2.46
Drug loading (%)	16.21 ± 0.22	16.06 ± 0.59
Entrapment yield (%)	87.89 ± 2.57	92.09 ± 6.99
Particle size, D(4,3) (µm)	40.498 ± 0.175	119.663 ± 1.295
Span value	1.440 ± 0.013	1.831 ± 0.034
Uniformity	0.425 ± 0.003	0.564 ± 0.010
Wall thickness (µm)	0.462 ± 0.074	0.411 ± 0.101

**Table 2:** Parameters of drug release kinetic models for free curcuminoids, cross-linked and non-cross-linked curcuminoids microcapsules (CPM).

Samples	Kinetic Models											Best Fit Model	t_50%_ (h)
	Zero Order		First Order		Higuchi		Hixson–Crowell		Korsmeyer–Peppas				
	R^2^	K_0_ (h^−1^)	R^2^	K_1_ (h^−1^)	R^2^	K_H_ (h^−1/2^)	R^2^	K_S_ (h^−1^)	R^2^	K (h^−n^)	*n*		
Free curcuminoids	0.9946	2.9106	0.9430	0.0486	0.8884	14.4569	0.8849	0.1487	0.9891	1.1382	1.3086	Zero order	17.18
Cross-linked CPM	0.9995	2.1975	0.9824	0.0309	0.9244	11.2619	0.8687	0.1216	0.9991	1.8510	1.0657	Zero order	22.75
Non-cross-linked CPM	0.9988	2.6294	0.9837	0.0396	0.9361	13.3776	0.8327	0.1377	0.9815	1.3283	1.3082	Zero order	19.02

Notes: R^2^ is the coefficient of determination; K_0,_ K_1_, K_H_, K_s_ and K are release rate constants; *n* is the release exponent; t_50%_ is the half-life of release.

**Table 3:** Values of coefficient of determination (R^2^), rate constant (k) and half-life of degradation (t_1/2_) for free curcuminoids and cross-linked curcuminoids microcapsule (CPM).

Samples	Zero-Order		First-Order		Second-Order		t_1/2_ (days)
	R^2^	k	R^2^	k	R^2^	k	
**Free curcuminoids**	0.9946	0.7504	0.9250	0.0081	0.9116	0.00010	66.6310
**Cross-linked CPM**	0.9278	0.2112	0.9248	0.0021	0.9216	0.00002	236.7400
